# Effect of Oxide Coating on Performance of Copper-Zinc Oxide-Based Catalyst for Methanol Synthesis via Hydrogenation of Carbon Dioxide

**DOI:** 10.3390/ma8115414

**Published:** 2015-11-16

**Authors:** Tetsuo Umegaki, Yoshiyuki Kojima, Kohji Omata

**Affiliations:** 1 Department of Materials & Applied Chemistry, College of Science & Engineering, Nihon University, 1-8-14, Kanda-Surugadai, Chiyoda-Ku, Tokyo 101-8308, Japan; kojima.yoshiyuki@nihon-u.ac.jp; 2 Department of Materials Science, Interdisciplinary Faculty of Science and Engineering, Shimane University, 1060, Nishikawatsu-Chou, Matsue, Shimane 690-8504, Japan; omata@riko.shimane-u.ac.jp

**Keywords:** oxide coating, copper-zinc oxide based catalyst, methanol synthesis, hydrogenation of carbon dioxide, hydrophilicity of oxides

## Abstract

The effect of oxide coating on the activity of a copper-zinc oxide–based catalyst for methanol synthesis via the hydrogenation of carbon dioxide was investigated. A commercial catalyst was coated with various oxides by a sol-gel method. The influence of the types of promoters used in the sol-gel reaction was investigated. Temperature-programmed reduction-thermogravimetric analysis revealed that the reduction peak assigned to the copper species in the oxide-coated catalysts prepared using ammonia shifts to lower temperatures than that of the pristine catalyst; in contrast, the reduction peak shifts to higher temperatures for the catalysts prepared using L(+)-arginine. These observations indicated that the copper species were weakly bonded with the oxide and were easily reduced by using ammonia. The catalysts prepared using ammonia show higher CO_2_ conversion than the catalysts prepared using L(+)-arginine. Among the catalysts prepared using ammonia, the silica-coated catalyst displayed a high activity at high temperatures, while the zirconia-coated catalyst and titania-coated catalyst had high activity at low temperatures. At high temperature the conversion over the silica-coated catalyst does not significantly change with reaction temperature, while the conversion over the zirconia-coated catalyst and titania-coated catalyst decreases with reaction time. From the results of FTIR, the durability depends on hydrophilicity of the oxides.

## 1. Introduction

Special attention has been paid to the sequestration of carbon dioxide by conversion to liquid compounds as a means of mitigating the environmental impact of this gas. In this context, the catalytic conversion of carbon dioxide to methanol via hydrogenation using heterogeneous catalysts has attracted enormous interest for its central role in carbon dioxide utilization [1,2,3]. Methanol can be used not only as the starting feedstock for many other useful chemicals but also as an alternative source in the production of liquid fuels. On an industrial scale, the production of methanol is generally achieved using Cu/ZnO/Al_2_O_3_ catalysts from synthesis gas, which is obtained via the steam reforming of natural gas and mainly contains carbon monoxide and hydrogen along with a small amount of carbon dioxide [4,5,6,7,8]. When there is no appreciable production of dimethyl ether, the reactions that this gas mixture undergoes are: the synthesis of methanol from carbon dioxide (Reaction (1)), the reverse water gas shift reaction, RWGS (Reaction (2)), and the “dry” methanol synthesis reaction from carbon monoxide (Reaction (3)) [9]:

CO_2_ + 3H_2_↔CH_3_OH + H_2_O Δ*H*_298 K_ = −49.58 kJ·mol^−1^ (1)

CO_2_ + H_2_↔H_2_O + CO Δ*H*_298 K_ = 41.12 kJ·mol^−1^ (2)

CO + 2H_2_↔CH_3_OH Δ*H*_298 K_ = −90.55 kJ·mol^−1^ (3)

The stability of the catalytic activity of the copper-based catalysts is affected by the feed containing a CO_2_/CO molar ratio more than unity. Furthermore, water is produced during methanol synthesis via carbon dioxide hydrogenation (Reaction (1)) and RWGS (Reaction (2)) accelerates the oxidation and deactivation of active sites, which is the influence of the water by-product produced via the hydrogenation of carbon dioxide [10,11,12,13,14]. The number of active sites decreases when inactive copper compounds, such as copper oxide, sintered active copper species, and copper with strongly adsorbed water molecules, are formed. Generally, mixing or coating the catalyst with inorganic materials such as silica is a promising technique to reduce the negative effect of water vapor [15,16,17].

The present study investigated the effect of oxide coating on catalytic activity and durability of a commercial copper-zinc oxide-based catalyst for the hydrogenation of carbon dioxide to methanol. The influence of preparation conditions such as the type of promoter (ammonia or L(+)-arginine) used for the sol-gel reaction for the coating oxide on the commercial catalyst was also investigated.

## 2. Results and Discussion

**Figure 1:**
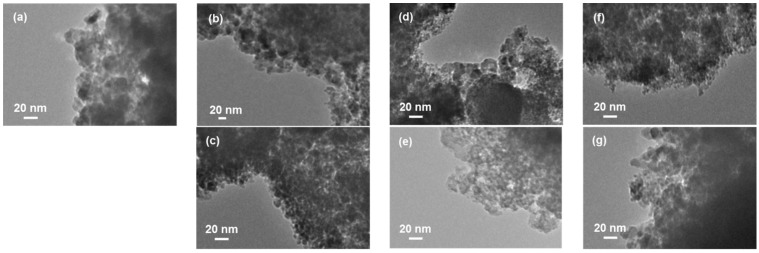
TEM images of pristine F04M catalyst (**a**) and F04M catalyst coated with silica (**b**,**c**), zirconia (**d**,**e**), and titania (**f**,**g**) using ammonia (**b**,**d**,**f**) and L(+)-arginine (**c**,**e**,**g**).

The morphologies of oxide-coated F04M catalysts prepared using various promoters were examined using TEM. The pristine F04M catalyst consists of finely dispersed particles with the diameters of approximately 20 nm (Figure 1a), while the oxide-coated catalysts prepared with ammonia consist of the particles of F04M catalyst loosely covered with silica with low contrast (Figure 1b,d,f). These results indicate that the F04M catalyst weakly bonded with the oxide coating with ammonia. However, the oxide-coated catalyst prepared with L(+)-arginine consisted of the accumulated fine particles of pristine F04M catalyst covered with a silica (Figure 1c,e,g). These results indicate that the F04M catalyst strongly bonded with the oxide coating with L(+)-arginine.

**Figure 2:**
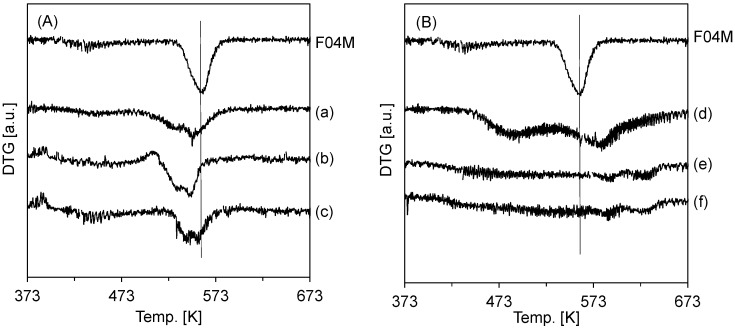
DTG curve registered upon H_2_-TPR of F04M catalyst coated with silica (a,d), zirconia (b,e), and titinia (c,f) using ammonia (**A**) and L(+)-arginine (**B**).

In order to obtain information about the reducibility of active copper species, temperature-programmed reduction (TPR) profiles observed upon treatment of the as-prepared samples in H_2_ are obtained by thermogravimetric analysis (TGA). Figure 2 shows the derivative thermogravimetry (DTG) curves of the as-prepared samples. The curves contained one main domain near 560 K. From the reduction profile of pristine F04M catalyst in Figure 2, clearly defined peaks assigned to copper species at around 560 K appeared in the DTG curve [18,19,20,21,22,23,24]. Compared with the result of the pristine F04M catalyst, the peak temperatures in the profile of the oxide-coated catalysts using ammonia were lower (Figure 2A), while the temperatures in the profile of oxide-coated catalysts using L(+)-arginine were higher (Figure 2B), and the reduction peak of the copper species of the oxide-coated catalysts was very broad. In other words, the temperatures depend on the strength of the bond between the oxide and F04M catalyst, and the bond strength of the samples prepared with L(+)-arginine was higher than that of the samples prepared with ammonia. Figure 2A,B also show temperature peaks at 553.6, 547.1, and 555.0 K for the catalysts coated with silica, zirconia, and titania using ammonia, respectively. On the other hand, the temperature peaks of the catalysts coated with silica, zirconia, and titania using L(+)-arginine were 585.0, 591.0, and 590.0 K, respectively. It is reported that reconstruction of the active copper particles occurred with the addition of TiO_2_ [25,26,27]. The result indicates that the active copper species in the TiO_2_-coated catalyst prepared using L(+)-arginine show broader dispersion than those in the pristine catalyst. These results indicate that the temperatures depend on the oxide coating over the catalyst.

**Figure 3:**
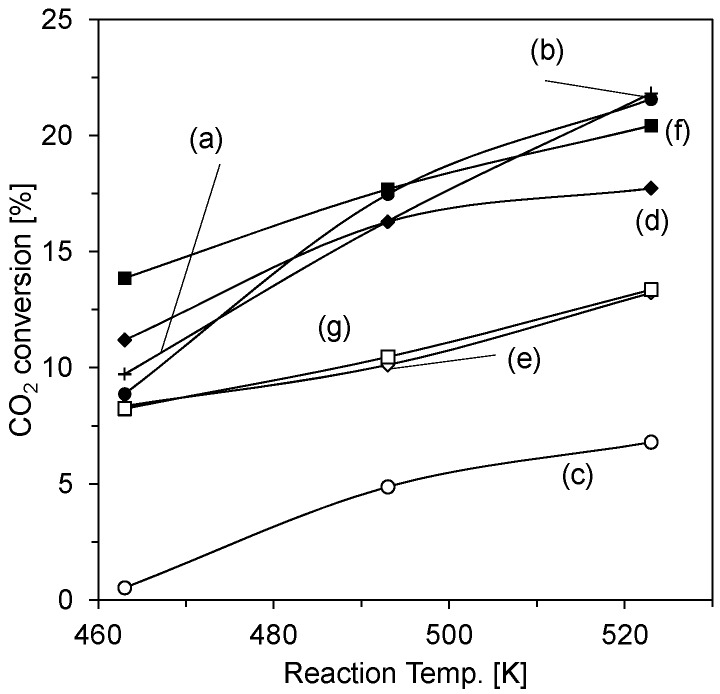
CO_2_ conversion over pristine F04M catalyst (a) and F04M catalyst coated with silica (b,c), zirconia (d,e), and titania (f,g) using ammonia (b,d,f) and L(+)-arginine (c,e,g). Reaction conditions: 3 MPa, W/F = 5 g·h^−1^·mol^−1^, H_2_/CO_2_/Ar = 72/24/4 (molar ratio).

Methanol synthesis from the hydrogenation of carbon dioxide was tested over the oxide-coated catalysts after pre-reduction procedures. Figure 3 shows the temperature dependence of the conversion of carbon dioxide over the oxide-coated catalysts. The conversion depends on the catalysts. In the entire temperature region, the conversion over the oxide-coated catalysts using ammonia was higher than that over the catalysts using L(+)-arginine. These results show that the bond strength between the oxide and the commercial catalyst influences the catalytic activity. 

Comparing the conversion among the oxide coatings, the silica-coated catalyst showed the highest activity in the high temperature region, while the zirconia-coated catalyst and titania-coated catalyst had high activity in the low temperature region. These results indicate that the temperature profile of the conversion depends on the nature of the catalyst’s oxide coating.

**Figure 4:**
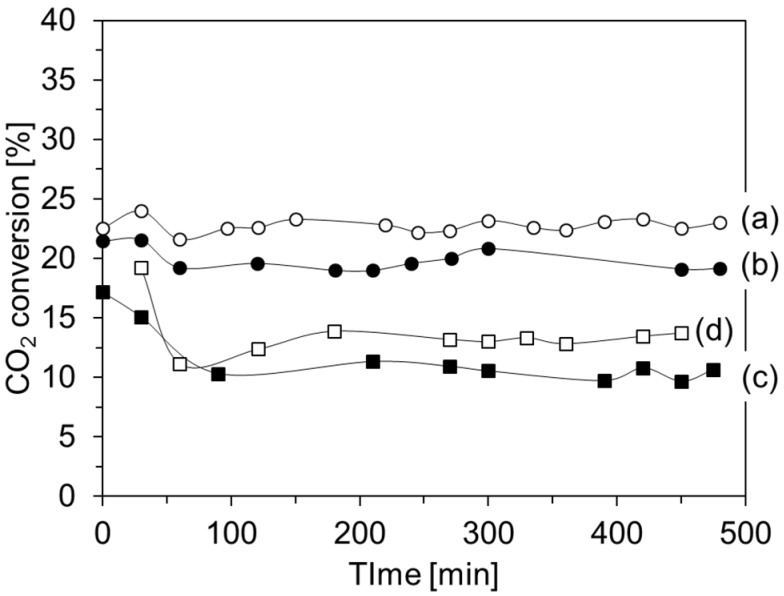
Time course of CO_2_ conversion over pristine F04M catalyst (a) and F04M catalyst coated with silica (b), zirconia (c), and titania (d). Reaction conditions: 523 K, 3 MPa, W/F = 5 g·h^−1^·mol^−1^, H_2_/CO_2_/Ar = 72/24/4 (molar ratio).

In order to verify the durability of the oxide-coated catalysts, they were tested for long reaction times. Figure 4 shows the conversion of carbon dioxide over the oxide-coated catalyst using ammonia. For comparison, the conversion over pristine F04M catalyst is also shown in this figure. The time course of the conversion depends on the catalyst’s oxide coating. The conversion over the silica-coated catalyst does not significantly change in terms of reaction time, while the conversion over the zirconia-coated catalyst and the titania-coated catalyst decrease up to 2 h before remaining constant up to 8 h. These results indicated that the silica-coated catalyst has a suitable activity for long reaction times. The silica-coated catalyst shows almost the same conversion per weight of F04 M (≈23%) compared with the F04M catalyst, and the silica-coated catalyst did not significantly reduce the catalytic activity. Table 1 shows product selectivity over the pristine F04M and the oxide-coated catalysts. As shown in Table 1, carbon monoxide and methanol are produced over all of the catalysts. Methanol selectivity over the silica-coated catalyst and the titania-coated catalyst was higher than that over the pristine F04M catalyst, while the selectivity over the zirconia-coated catalyst was the same as that over the pristine F04M catalyst. These results indicated that silica and titania coatings on the F04M catalyst effectively improved methanol selectivity. It is reported that the addition of TiO_2_ improves the methanol selectivity because of the reconstruction of the active copper particles and/or its acid-base property, which controls the stability of the reaction intermediate [25,26,27].

**Table 1:** Product selectivity over the various catalysts.

Catalyst	CO Selectivity (%)	CH_3_OH Selectivity (%)
F04M	62.1	37.9
SiO_2_ coat	53.1	43.9
ZrO_2_ coat	61.7	38.3
TiO_2_ coat	45.3	54.7

Reaction conditions: 523 K, 3 MPa, W/F = 5 g·h^−1^·mol^−1^, H_2_/CO_2_/Ar = 72/24/4 (molar ratio).

**Figure 5:**
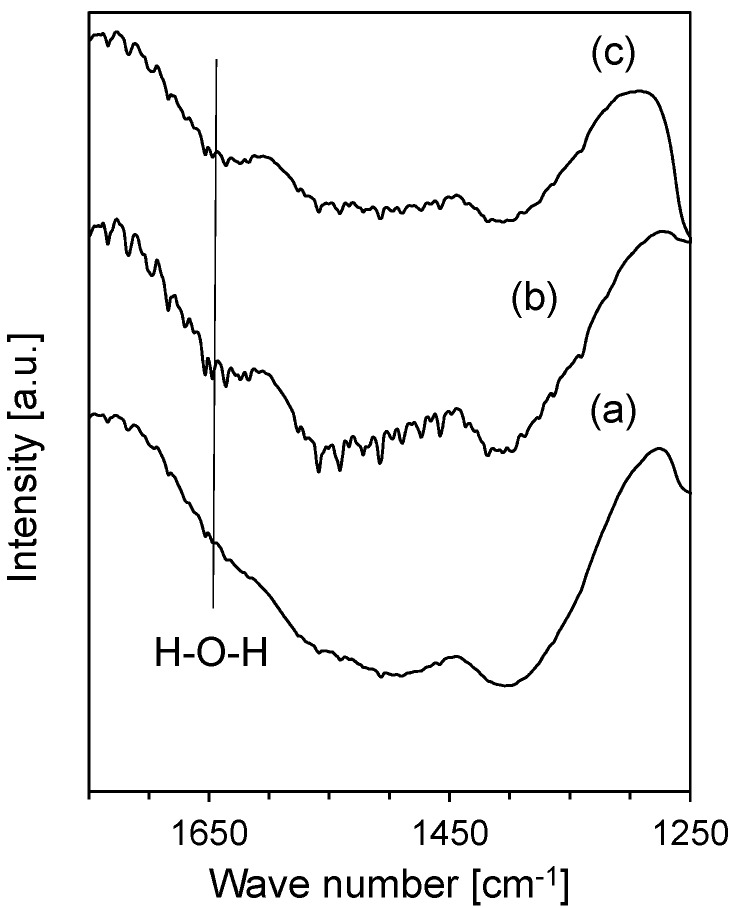
FTIR spectra of F04M catalyst coated with silica (a), zirconia (b), and titania (c).

In order to determine differences in activity, the oxide-coated catalysts were characterized by FTIR spectroscopy to assess their hydrophilicity. Figure 5 shows the FTIR spectra for the oxide-coated catalysts prepared with ammonia. The zirconia-coated catalyst and the titania-coated catalyst displayed a band of the H–O–H bond at around 1640 cm^−1^ [28], while the silica-coated catalyst lacked this band. These results indicated that the silica-coated catalyst had a relatively low hydrophilicity compared with the zirconia-coated catalyst and the titania-coated catalyst because the oxide with low hydrophilicity probably reduced the influence of the steam by-product. Therefore, the silica-coated catalyst had a higher durability than the zirconia-coated catalyst or titania-coated catalyst.

## 3. Experimental Section

### 3.1. Catalyst Preparation

Various oxides (silica, zirconia, titania) were coated on commercial copper-zinc oxide-based catalyst (JGC Catal. Chem. Ltd.; Kanagawa, Japan, F04M) by a sol-gel method. An aqueous solution of ammonia (Kanto Chemical Co., Tokyo, Japan, 28 wt % solution, >99.0%) or L(+)-arginine (Wako Chemical Co., Osaka, Japan, >99.0%) were mixed with 0.5 g of F04M in ethyl alcohol, then tetraethoxysilane (Wako Pure Chem. Co., Osaka, Japan, >99.0%), zirconium butoxide (Sigma-Aldrich Co. St. Louis, MO, USA, LLC., 80 wt % solution in 1-butanol), or titanium-*n*-butoxide (Wako Pure Chem. Co., Osaka, Japan, >97.0%) was added to the solution, followed by stirring at 323 K for 3 h and centrifuging at a rotation speed of 6000 rpm for 5 min.

### 3.2. Characterization

The morphology of the oxide-coated catalysts was observed using a Hitachi FE2000 (Hitachi High-Tech. Co., Tokyo, Japan) transmission electron microscope (TEM) operated at an acceleration voltage of 200 kV. The temperature-programmed reduction–thermogravimetric analyses (TPR–TGA) were performed with a Rigaku TG8120 (Rigaku Co., Tokyo, Japan) instrument. TPR profiles were recorded by passing a 10 vol % H_2_ in Ar (260 mL·min^−1^) through the sample (~2 mg) heated at a constant rate of 5 K·min^−1^ up to 1173 K.

### 3.3. Catalytic Activity

The catalysts were evaluated in a tubular stainless steel, fixed-bed reactor (1/2′′ i.d) equipped with a temperature-programmed control unit and a K-type thermocouple. Each sample was loaded between two layers of quartz wool in the reactor. Before experiment, the catalyst was firstly pre-reduced at 553 K and atmospheric pressure in a hydrogen stream (1 vol % H_2_ in Ar) and cooled to desired temperature. Subsequently, synthesis gas (H_2_/CO_2_/Ar = 72/24/4 (molar ratio)) was introduced at W/F = 5 g·h^−1^·mol^−1^, at a pressure of 3.0 MPa using a mass flow controller (Brookhaven). The effluent gases were analyzed by two GC-TCD (Shimadzu GC-8A, Shincarbon ST column and Shimadzu GC-8A, FlusinT column). Conversion and selectivity were calculated by internal standard and mass-balance methods. CO_2_ conversion was calculated as follow:

CO_2_ conversion = 1 − F[CO_2_]_out_/F[CO_2_]_in_

Maximum error margin of the conversion in this study was ± 7%.

## 4. Conclusions

This study investigated the effect of oxide coatings on the activity of copper-zinc oxide-based catalyst for methanol synthesis via the hydrogenation of carbon dioxide. A commercial copper-zinc oxide-based catalyst was coated with various oxides (silica, zirconia, titania) by a sol-gel based method. In this study, the influence of preparation conditions such as kinds of promoters (ammonia or L(+)-arginine) on the sol-gel reaction was investigated. From the result of TPR-TGA, the reduction peak assigned to the copper species of the oxide-coated samples prepared using ammonia shifted to lower temperatures than that of the pristine F04M catalyst, while the copper reduction peaks shifted to higher temperatures for the oxide-coated samples prepared using L(+)-arginine, indicating that the copper species that were more weakly bonded with oxide were more easily reduced. The coated catalyst prepared with ammonia shows a higher conversion of carbon dioxide than the coated catalyst prepared with L(+)-arginine, indicating that the catalyst with a weaker oxide bond has a higher activity than the catalyst with a strong oxide bond. Among the oxide-coated catalysts prepared using ammonia, the silica-coated catalyst displayed the highest activity in the high reaction temperature region, while the zirconia-coated and titania-coated catalysts had high activities at low reaction temperatures. At high reaction temperatures, the conversion over the silica-coated catalyst did not significantly change with the reaction time, while the conversion over the zirconia-coated and titania-coated catalysts decreased during the initial reaction time, indicating that the silica-coated catalysts have a more suitable activity for long reaction times. The zirconia-coated catalyst and the titania-coated catalyst displayed the band of the H–O–H bond, while the silica-coated catalyst did not display the band, indicating that the silica-coated catalyst had a relatively low hydrophilicity compared with the zirconia-coated and the titania-coated catalysts. The silica-coated catalyst, therefore, had a high durability compared with the other oxide-coated catalysts.
